# Pharmacokinetics and Dose Optimization of Ethambutol in Children on First-Line Antituberculosis Therapy: An Individual Patient Data Meta-Analysis

**DOI:** 10.1093/infdis/jiag194

**Published:** 2026-03-30

**Authors:** Tae Kyu Chung, Eunsol Yang, Maureen Shin, Belen P Solans, Xujia Zhou, Sejung Hwang, Michael C Yoon, Dvijen Purohit, Dongsheng Yang, Samer Mouksassi, Agathe Béranger, Adrie Bekker, Awewura Kwara, Navarat Panjasawatwong, Radojka M Savic

**Affiliations:** Department of Bioengineering and Therapeutic Sciences, Schools of Pharmacy and Medicine, University of California, SanFrancisco, San Francisco, California, USA; Center for Tuberculosis, University of California, SanFrancisco, San Francisco, California, USA; Department of Bioengineering and Therapeutic Sciences, Schools of Pharmacy and Medicine, University of California, SanFrancisco, San Francisco, California, USA; Center for Tuberculosis, University of California, SanFrancisco, San Francisco, California, USA; Department of Bioengineering and Therapeutic Sciences, Schools of Pharmacy and Medicine, University of California, SanFrancisco, San Francisco, California, USA; Center for Tuberculosis, University of California, SanFrancisco, San Francisco, California, USA; Department of Bioengineering and Therapeutic Sciences, Schools of Pharmacy and Medicine, University of California, SanFrancisco, San Francisco, California, USA; Center for Tuberculosis, University of California, SanFrancisco, San Francisco, California, USA; Department of Bioengineering and Therapeutic Sciences, Schools of Pharmacy and Medicine, University of California, SanFrancisco, San Francisco, California, USA; Center for Tuberculosis, University of California, SanFrancisco, San Francisco, California, USA; Department of Bioengineering and Therapeutic Sciences, Schools of Pharmacy and Medicine, University of California, SanFrancisco, San Francisco, California, USA; Center for Tuberculosis, University of California, SanFrancisco, San Francisco, California, USA; Department of Bioengineering and Therapeutic Sciences, Schools of Pharmacy and Medicine, University of California, SanFrancisco, San Francisco, California, USA; Center for Tuberculosis, University of California, SanFrancisco, San Francisco, California, USA; Department of Bioengineering and Therapeutic Sciences, Schools of Pharmacy and Medicine, University of California, SanFrancisco, San Francisco, California, USA; Center for Tuberculosis, University of California, SanFrancisco, San Francisco, California, USA; Department of Bioengineering and Therapeutic Sciences, Schools of Pharmacy and Medicine, University of California, SanFrancisco, San Francisco, California, USA; Center for Tuberculosis, University of California, SanFrancisco, San Francisco, California, USA; Department of Bioengineering and Therapeutic Sciences, Schools of Pharmacy and Medicine, University of California, SanFrancisco, San Francisco, California, USA; Center for Tuberculosis, University of California, SanFrancisco, San Francisco, California, USA; Department of Bioengineering and Therapeutic Sciences, Schools of Pharmacy and Medicine, University of California, SanFrancisco, San Francisco, California, USA; Center for Tuberculosis, University of California, SanFrancisco, San Francisco, California, USA; Université Paris Cité, INSERM, UMR1343, Pharmacologie et Évaluation des Thérapeutiques Chez L'Enfant et la Femme Enceinte, Paris, France; Réanimation et Soins Intensifs Polyvalents Pédiatriques—SMUR Pédiatrique, AP-HP.Centre, Hôpital Necker-Enfants Malades, Paris, France; Department of Paediatrics and Child Health, Stellenbosch University, Cape Town, South Africa; Division of Infectious Diseases and Global Medicine, University of Florida College of Medicine, Gainesville, Florida, USA; Department of Pharmaceutical Care, Faculty of Pharmacy, Payap University, Chiang Mai, Thailand; Department of Bioengineering and Therapeutic Sciences, Schools of Pharmacy and Medicine, University of California, SanFrancisco, San Francisco, California, USA; Center for Tuberculosis, University of California, SanFrancisco, San Francisco, California, USA

**Keywords:** individual patient data meta-analysis, population pharmacokinetics, tuberculosis, pediatrics, global health

## Abstract

**Background:**

Tuberculosis affects over one million children annually. Ethambutol (EMB) is a key component of first-line therapy, but comprehensive pediatric pharmacokinetic studies have been lacking.

**Methods:**

EMB concentrations and individual-level patient data from 3 studies were analyzed using nonlinear mixed effects modeling. Simulations compared current World Health Organization (WHO)–recommended doses and model-informed optimized doses. Target exposure distribution (area under the curve, 13.8–17.7 mg·h/L), which had been previously established in adult clinical trials, guided dose optimization.

**Results:**

A total of 220 participants (0.2–15 years; 3.6 and 43.0 kg) contributed 1085 plasma EMB concentrations for model development. Weight effects were incorporated for clearance (CL), intercompartmental CL, and volumes of distribution in central and peripheral compartments. Age affected CL through a maturation function. Simulations indicated children weighing <25 kg had median exposures below target exposure distribution at current WHO-recommended doses. Optimized weight-band dosing would increase doses in approximately 40.5% of the patients, with an additional 5% potentially benefiting.

**Conclusions:**

Our individual patient data meta-analysis supports the revision of current pediatric EMB dosing guidelines for the treatment of tuberculosis. Our findings aim to ensure adequate drug exposure across all pediatric weight bands, while also accounting for toxicity and challenges related to formulation implementation and adherence.

## Teaser

An individual patient data meta-analysis of ethambutol in children showed that current World Health Organization dosing underdoses children, particularly those who are malnourished. Model-informed weight-band dosing improved target attainment, supporting optimized regimens to ensure therapeutic exposures across pediatric populations.

Tuberculosis affects 1.3 million children (95% uncertainty interval, 1.2–1.3 million) annually worldwide, accounting for 12% of all tuberculosis cases globally [1, 2]. In 2023 alone, 166 000 children died of tuberculosis, representing 15% of all tuberculosis-related deaths [2]. Although delayed or missed diagnoses are the leading cause of tuberculosis-related death in children, clinical evidence shows that even with timely diagnosis and treatment, tuberculosis outcomes remain suboptimal in countries with a high tuberculosis burden [3, 4]. This may be partly due to subtherapeutic drug exposures resulting from inadequate dosing strategies in children, who have distinct pharmacokinetic (PK) profiles compared with adults [5, 6]. Characterizing the PKs of tuberculosis medications and optimizing dosing regimens is critical to maximizing treatment benefit among pediatric populations.

Current World Health Organization (WHO) guidelines recommend including ethambutol (EMB) during the first 2 months of first-line treatment regimens [1]. EMB plays a role in inhibiting mycobacterial growth and preventing the emergence of resistance to coadministered first-line treatment medications [6]. This recommendation is particularly important for individuals with extensive disease or living in settings with a high prevalence of human immunodeficiency virus (HIV) or isoniazid resistance, where treatment typically spans 4–6 months. EMB-associated ocular toxicity is clearly dose and exposure related in adults, whereas in children it appears rare; nevertheless, EMB should be administered with caution for pediatric population [6]. The current recommended doses of EMB in children are 15–25 mg/kg across all weight bands [1, 6]. However, these dosing recommendations were developed without fully incorporating factors such as maturation of organ size or nutritional status, both of which significantly influence the extent of drug metabolism and disposition [7].

In adults, EMB exhibits relatively well-characterized PKs. It has low plasma protein binding (median, 12%; range, 4.0%–24.0%), suggesting that variations in plasma protein concentrations are unlikely to substantially affect systemic drug exposure [8]. EMB is primarily eliminated unchanged via renal excretion through glomerular filtration, making renal function the principal determinant of systemic clearance (CL) [9], and food intake has not been shown to have a clinically meaningful effect on the extent of absorption [10]. While these properties indicate relatively predictable PK characteristics in adults, extrapolation to pediatric populations remains uncertain due to developmental changes in renal maturation, body composition, and growth-related physiological processes.

Supporting this concern, EMB exposures were suboptimal for the majority of children in clinical studies conducted in India and Malawi under the current dosing regimen [5, 11, 12]. These findings highlight the urgent need for comprehensive PK studies that account for age-related maturation and nutritional factors to optimize dosing strategies, ensuring both efficacy and safety in these vulnerable populations. In particular, safety concerns related to ocular toxicity from high EMB exposure must be carefully considered, as this dose-dependent adverse effects can be difficult to detect in very young children, especially in resource-limited settings [6, 13–15]. In addition, the EMB dosing table should be revised following the harmonized WHO weight-band framework (Harmonized Weight Band section in the Supplementary Materials) to enhance alignment with global treatment guidelines and support more practical implementation [16].

Previous population PK analyses of EMB in pediatric populations have consistently demonstrated that the current WHO-recommended dosing is insufficient to achieve target exposures in the smallest children [17–19]. However, these analyses did not fully explore the effects of nutritional status and other clinically relevant covariates on EMB PKs. Therefore, a more comprehensive individual patient data meta-analysis (IPDMA) incorporating additional data sources and covariate information is warranted to further characterize EMB PKs across a heterogeneous pediatric population and to support refined and evidence-based dosing recommendations. The primary objectives were to (1) perform an IPDMA by developing a population PK model that characterizes EMB PK profiles in pediatric populations and (2) assess EMB exposure across harmonized weight bands to inform optimized dosing strategies.

## METHODS

### Clinical Studies and IPDMA Dataset

We conducted a literature search to identify studies reporting EMB PKs in pediatric populations, for any indication, for inclusion in the analysis. Additional methods for identifying eligible studies are described in the Supplementary Materials (Supplementary Tables 1 and 2). The primary authors of eligible studies were contacted to request available individual-level data, including demographics (eg, age, sex, weight, height, and race), medical history (eg, HIV status), concomitant mediation use, EMB administration details (eg, dose, dosing time, formulation, route of administration, and PK sampling times), and plasma EMB concentration values. All included studies were conducted with appropriate ethical approval, and data sharing agreements were established for studies contributing individual participant data. All shared data were fully deidentified prior to analysis.

### Population PK Analyses

EMB concentrations were pooled and analyzed using nonlinear mixed-effects modeling. One- and 2-compartment disposition models were evaluated with first-order absorption and both linear and nonlinear elimination. More detailed modeling methods are described in the Base Model Development and Covariate Model Development sections of the Supplementary Materials.

To evaluate the effect of organ maturation in pediatric populations, a sigmoidal maximum response $\left(E_{max}\right)$ model—characterized by an initial slow growth phase followed by a faster growth phase and an eventual plateau—was used (Equation 1) [20]. The relationship is given by $P_{i,cov}=P_{p}\;F_{mf}$, where $P_{i,cov}$ represents the PK parameter for the *i*^th^ individual after incorporating the covariate effect, $P_{p}$ represents the typical population value, and


(1) $$F_{mf}=\frac{PMA^{Hill}}{PMA^{Hill}+PMA_{50}^{Hill}}.$$

In this equation, $F_{mf}$ represents the maturation function, PMA represents the postmenstrual age (ie, PMA=((Age+0.001)⋅52)+40), Hill is the coefficient that determines the steepness of the maturation curve, and PMA_50_ is the postmenstrual age at which 50% of the adult PK parameter value is reached.

The final population PK model was considered robust if the estimated PK parameter estimates were consistent with the medians of the PK parameter estimates obtained from 500 bootstrap replicates using the original dataset. In addition, prediction-corrected visual predictive check (pcVPC) plots were performed for the final population PK model, stratified by studies, to assess potential model misspecification or bias. The pcVPC were constructed using 500 simulated datasets generated from the final population PK model. The 2.5th percentile, median, and 97.5th percentile of the prediction-corrected simulated concentrations were compared with those of the observed plasma EMB concentrations.

### Simulation of EMB Exposures for Dose Optimization

Monte-Carlo simulations were performed 1000 times using the final population PK model to investigate EMB exposures. The simulation dataset included all demographic characteristics from the EMB model development dataset, as well as pediatric demographic data stored in house from tuberculosis clinical trials and datasets from the Demographic and Health Surveys Program spanning 30 countries (Supplementary Figure 5) [21]. The individual-level patient data from the model development dataset were used to reflect the distribution of significant covariates. Steady-state 24-hour exposure (24-hour area under the curve at steady state [AUC_24,ss_]) was calculated, per individual, using Equation 2:


(2) $$AUC_{24,ss}=\frac{Dose\;\;\;Bioavailability}{Clearance}.$$

Simulated EMB exposures under the current WHO dosing regimen were used to identify the distribution and proportion of individuals currently achieving exposures within both the therapeutic window (ie, 11.0–18.0 mg·h/L [Alkhafaji S, Yang E, Chang VK, et al. Ethambutol population pharmacokinetics and pharmacodynamics from a phase 3 confirmatory trial for the treatment of tuberculosis; in press]) and the distributions of exposures (interquartile range, 13.8–17.7 mg·h/L [22],) to determine optimal doses that fall within the target range using harmonized weight bands (Supplementary Table 3). The interquartile range of EMB exposure was calculated using the individual PK data from Study 31/A5349, the largest and most diverse phase III clinical study to date for tuberculosis medications [22, 23], as we had access to these data. Target exposure distribution, which had been previously established in adult clinical trials, guided dose optimization. To determine the optimal dose for each weight band, factors such as available formulations of EMB and the number of tablets required for each dose were also considered along with potential adverse events related to higher exposures, to ensure ease of administration and address safety concerns, respectively.

## RESULTS

### Participants

Three studies were included in the analysis: 1 each conducted in South Africa, Ghana, and Vietnam (Supplementary Table 1) [13, 17, 18]. Studies conducted by Bekker et al [13] and Horita et al [17] have been registered at CliniclaTrials.gov under registration numbers NCT01637558 and NCT01687504, respectively. Three studies were conducted in South Africa, Ghana, and Vietnam (Table 1). EMB was swallowed by mouth, except in the study by Bekker et al [13], where participants received EMB through nasogastric tubes, as they were all <1 year old. PK sampling was conducted after ≥2 weeks of treatment by Bekker et al [13], on both the first day and at steady state in Panjasawatwong et al [18], and only at steady state by Horita et al [17]. All 3 studies used dispersible fixed-dose combination (FDC) tablets, dosed at 15–25 mg/kg/d (Table 1).

**Table 1.** Details of Studies Included in Individual Patient Data Meta-analysis of Ethambutol

Study Details	Bekker et al (2016) [13]	Horita et al (2018) [17]	Panjasawatwong et al (2020) [18]
Study description	Observation cohort study with intensive PK sampling among infants with or without HIV	Prospective, observational, intensive PK study in children with or without HIV	Prospective descriptive study in children with tuberculosis meningitis
Country	South Africa	Ghana	Vietnam
Sample size	16	113	100
Dosing regimen	15–25 mg/kg/d for 2 mo	15–25 mg/kg/d for 2 mo	15 mg/kg/d for 2 mo
Formulation	Dispersible FDC tablet (RIF, INH, PZA) ± EMB	Dispersible FDC tablet (RIF, INH, PZA, EMB)	Dispersible FDC tablet (INH, RIF, PZA) + EMB (oral), and streptomycin (intramuscular)
Lower limit of quantification	0.08 mg/L	0.08 mg/L	0.08 mg/L
Timing of sampling	Week 2 or later: before and 1, 2, 4, 6, and 8 h after dose	Week 4 or later: before and 1, 2, 4, and 8 h after dose	Days 1 and 14: 2 samples randomly obtained 1, 2, 3, 4, 5, 6, 8, 12, 18, or 24 h after dose; days 30 and 90: 1 sample randomly obtained 3, 4, or 5 h after- dose

Abbreviations: EMB, ethambutol; FDC, fixed-dose combination; HIV, human immunodeficiency virus; INH, isoniazid; PK, pharmacokinetic; PZA, pyrazinamide; RIF, rifampicin.

A total of 229 participants were included from these studies; however, data from 9 participants were excluded from the analysis due to missing or implausible covariate values (eg, extremely high or low body mass index [BMI], potentially due to data entry errors in weight or height). In addition, 68 samples (5.9%) were below the level of quantification and were excluded from the analysis [24]. As a result, a total of 220 participants and their corresponding 1085 plasma concentrations of EMB were included in the final IPDMA dataset and analyses (Table 2 and Supplementary Figure 1). The participants ranged in age from 2.0 months to 15 years and in weight from 3.6 to 43 kg, with a median age of 4 years and a median weight of 12.5 kg (Table 2 and Supplementary Figures 1 and 3). Overall, 27.7% of participants were living with HIV. The median weight-for-age, height-for-age, weight-for-height, and BMI-for-age *z* scores were <0 across all studies, reflecting the high prevalence of undernutrition and stunting commonly observed in pediatric tuberculosis populations and supporting the relevance of the dataset for evaluating nutrition-related PK variability (Supplementary Figure 3).

**Table 2.** Demographic and Clinical Characteristics of Children Included in the Dataset

Characteristic	Bekker et al (2016) [13] (n = 14)	Horita et al (2018) [17] (n = 108)	Panjasawatwong et al (2020) [18] (n = 98)	Total (n = 220)
Age group, no. (%)				
<6 mo	5 (35.7)	2 (1.9)	7 (7.1)	14 (6.4)
6 mo to 1 y	9 (64.3)	8 (7.4)	12 (12.2)	29 (13.2)
1–5 y	0 (0.0)	41 (38.0)	45 (45.9)	86 (39.1)
5–10 y	0 (0.0)	37 (34.3)	18 (18.4)	55 (25.0)
≥10 y	0 (0.0)	20 (18.5)	16 (16.3)	36 (16.4)
Male sex, no. (%)	10 (71.4)	59 (54.6)	53 (54.6)	123 (55.9)
Age, median (range)	0.5 y (3.4 mo to 1.0 y)	5.0 y (5.0 mo to 14.0)	3.0 y (2.0 mo to 15.0)	4.0 y (2.0 mo to 15.0 y)
Weight, median (range), kg	6.7 (4.4–9.7)	14.3 (3.6–40.9)	11.0 (4.0–43.0)	12.5 (3.6–43.0)
Height, median (range), cm	63.5 (51.3–72.2)	100.0 (58.0–161.0)	87.0 (48.0–170.0)	93.0 (48.0–170.0)
Nutritional status (*z* scores), median (range)^a^				
WAZ score	−2.2 (−4.5 to 0.2)	−1.9 (−5.8 to 2.2)	−1.7 (−5.3 to 2.0)	−2.1 (−5.8 to 2.2)
HAZ score	−2.6 (−5.4 to 1.3)	−1.8 (−5.9 to 5.1)	−1.6 (−5.6 to 2.2)	−1.7 (−5.9 to 5.1)
WHZ score^b^	−0.5 (−3.1 to 2.2)	−1.6 (−5.0 to 2.6)	−0.8 (−3.5 to 3.4)	−1.0 (−5.0 to 3.4)
BAZ score	−0.7 (−3.1 to 4.9)	−1.5 (−4.9 to 3.0)	−1.4 (−4.5 to 2.8)	−1.3 (−4.9 to 4.9)
Malnourished, no. (%)^c^	8 (57.1)	72 (66.7)	69 (70.4)	149 (67.7)
Living with HIV, no. (%)	2 (14.3)	55 (50.9)	4 (4.1)	61 (27.7)

Abbreviations: BAZ, body mass index–for–age *z*; HAZ, height-for-age *z*; HIV, human immunodeficiency virus; WAZ, weight-for-age *z*; WHZ, weight-for-height *z*.

^a^Except where otherwise noted, *z* scores were calculated for participants <10 years old. Two, 5, and 2 patients from the studies by Bekker et al [13], Horita et al [17], and Panjasawatwong et al [18], respectively, were excluded from the dataset due to missing or implausible covariate values (eg, extremely high or low body mass index, potentially due to data entry errors for weight or height).

^b^Calculated for those <5 years old.

^c^Malnourishment was defined as defined as BAZ, HAZ, WAZ, or WHZ scores below −2.

### Population Pharmacokinetic Model

The final structural model was a 2-compartment model with a 1-transit compartment for absorption that adequately described the concentration time profile of EMB (Supplementary Figure 6; control stream available in the Supplementary Materials). The interindividual variability (IIV) in the volume of distribution in the central compartment (Vc) was high, with a coefficient of variation of 71.4% (relative standard error [RSE], 41.0%), whereas the IIV for CL and mean transit time were low, at 19.3% (RSE, 53.0%) and 22.1% (RSE, 3.0%), respectively. A proportional error of 35.9% (RSE, 13.0%) was estimated to describe the residual variability. The IIV was found to be significant when added to CL, Vc, and mean transit time. None of the interoccasional variability or interstudy variability terms improved the model fit or reduced the respective IIV, except for interoccasional variability in bioavailability (ie, F1).

Among all covariates tested, the following were found to be significant: the effects of weights on CL, intercompartment CL (Q), Vc, and the volume of distribution in the peripheral compartment, as well as the age effect through a maturation function for CL. The extent of the weight effect on CL did not differ between malnourished and well-nourished participants when the Hill coefficients were estimated separately. In addition, because the estimated Hill coefficients were comparable to allometric scaling and the bootstrap confidence interval included 0.75 for CL and Q and 1 for Vc and the volume of distribution in the peripheral compartment, these values were fixed in the final model. Most PK parameters were precisely estimated, except for the power term (ie, the Hill coefficient) of the maturation function, which was estimated at 7.8 and exhibited a relatively wide 95% confidence interval (ie, 2.0–20.5). Therefore, we fixed the Hill coefficient to 3.25, consistent with the value fixed in a previously published population PK model [25].

The inclusion of age through a maturation function for CL improved the model fit, and the estimated postmenstrual age at which 50% maturation is reached was 9.7 months (42.1 weeks; Table 3). This was comparable to the previously reported values for the PMA_50_ if glomerular filtration rate maturation is reached [26, 27]. Participants living with HIV (27.7%) and those with HIV did not have significantly different CLs. Furthermore, the use of a nasogastric tube did not have a significant effect on absorption or bioavailability. No prediction bias was observed based on the goodness-of-fit plots (Supplementary Figure 8).

**Table 3.** Parameters of the Final Individual Patient Data Meta-analysis Model for Ethambutol

EMB Parameter	Typical Value (RSE, %)	Bootstrap Result Median (95% CI)^a^	Variability, CV, % (RSE, %)		Shrinkage, %
			IIV	IOV	
Typical values					
CL, L/h	30.3 (6.0)	30.4 (28.5–32.3)	19.3 (53.0)	…	45.0
MTT, h	2.0 (81.0)	2.0 (1.8–2.2)	22.1 (3.0)	…	55.0
Q, L/h	23.2 (67.0)	23.2 (20.6–25.8)	…	…	…
Vc, L	61.3 (140.0)	62.3 (52.2–74.7)	71.4 (41.0)	…	31.0
Vp, L	148.0 (60.0)	149.4 (129.1–173.9)	…	…	…
F1	1 FIX	1 FIX	…	39.9 (38.0)	47.0
Proportional RUV, %	35.9 (13.0)	35.9 (33.4–38.8)	…	…	…
Covariate effects					
Power term, standardized weight effect with CL and Q^b,c^	0.75 FIX	0.75 FIX	…	…	…
Power term, standardized weight effect with Vc and Vp^d,e^	1 FIX	1 FIX	…	…	…
Power term, Hill coefficient for CL^f^	3.25 FIX	3.25 FIX	…	…	…
PMA_50_ for CL, wk^b^	42.1 (16.0)	42.2 (20.8–52.2)	…	…	…

Abbreviations: CI, confidence interval; CL, clearance; CV, coefficient of variation; FIX, parameter fixed, ; IIV, interindividual variability; Hill, coefficient that determines the steepness of the maturation curve; IOV, interoccasional variability; MTT, mean transit time; PMA, postmenstrual age; PMA_50_, postmenstrual age at 50% of maturation; Q, intercompartment CL of unbound ethambutol; RSE, relative standard error; RUV, residual unexplained variability; Vc, volume of distribution in the central compartment; Vp, volume of distribution in the peripheral compartment.

^a^The 95% CIs were derived using 500 bootstrap runs.

^b^ $\frac{\mathrm{CL}}{F}=\;\theta_{\;\mathrm{pop}}\cdot {\left(\frac{\mathrm{Weight}}{12.5}\right)}^{0.75}\cdot \left(\frac{\mathrm{PM}A^{\mathrm{Hill}}}{\mathrm{PM}A_{50}^{\mathrm{Hill}}+\mathrm{PM}A^{\mathrm{Hill}}}\right)$ .

^c^ $\frac{Q}{F}=\;\theta_{\;\mathrm{pop}}\;\cdot {\left(\frac{\mathrm{Weight}}{12.5}\right)}^{0.75}$ .

^d^ $\frac{\mathrm{Vc}}{F}=\;\theta_{\;\mathrm{pop}}\;\cdot {\left(\frac{\mathrm{Weight}}{12.5}\right)}^{1}$ .

^e^ $\frac{\mathrm{Vp}}{F}=\;\theta_{\;\mathrm{pop}}\cdot {\left(\frac{\mathrm{Weight}}{12.5}\right)}^{1}$ .

^f^The Hill coefficient describes the steepness of the maturation function.

**Figure 1.**
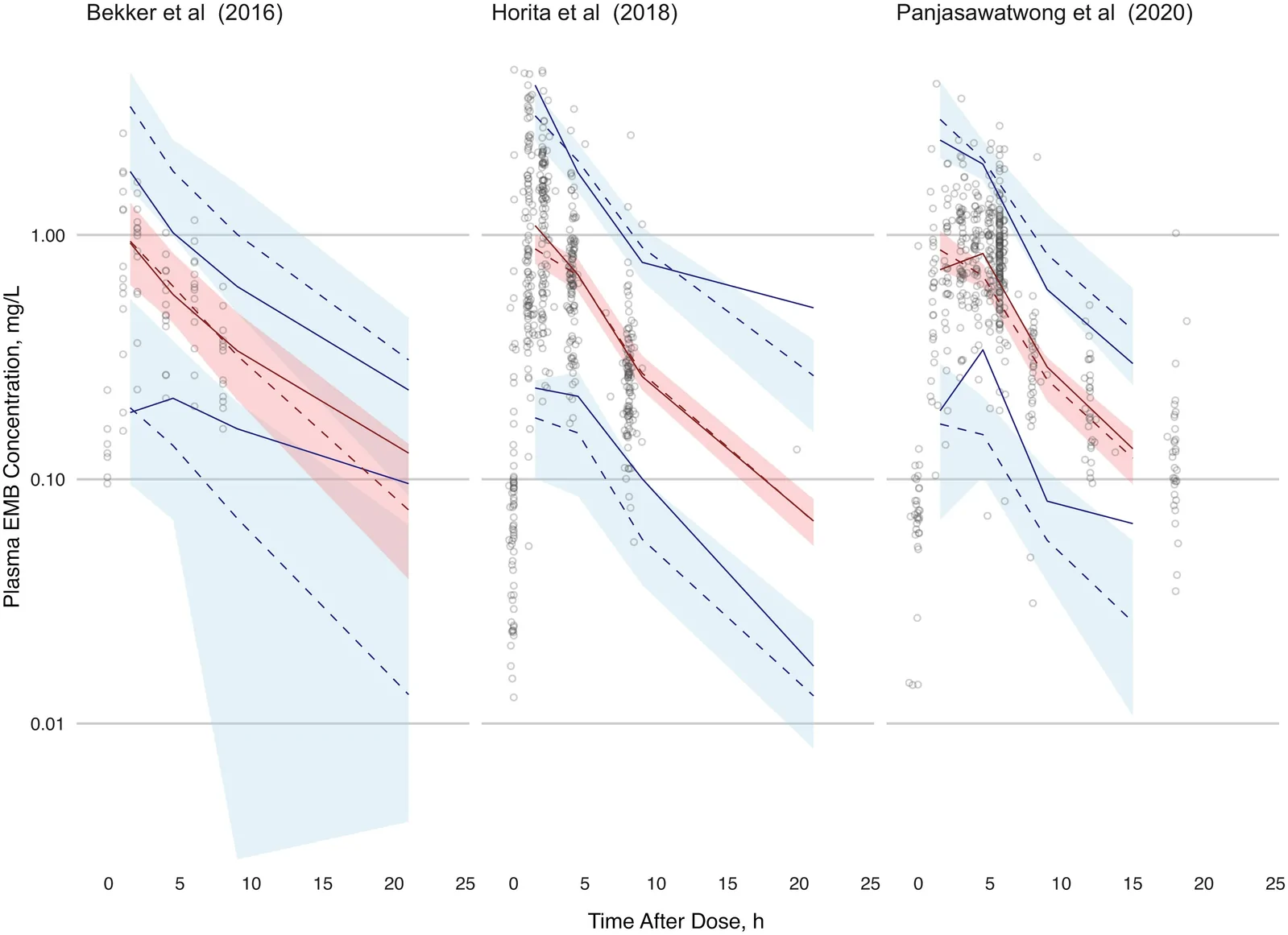
Prediction-corrected visual predictive check plots of the final individual patient data meta-analysis model for ethambutol (EMB). Open circles represent the observed EMB concentrations; solid and dashed red lines, median values of the observed and predicted plasma EMB concentrations, respectively; solid and dashed blue lines, lower 2.5th and 97.5th values of the observed and predicted plasma EMB concentrations; and shaded areas, 95% confidence intervals around the lower 2.5th (blue), median (red), and upper 97.5th (blue) predicted EMB concentrations. The vertical axis for the EMB concentrations is shown in the logarithmic scale.

**Figure 2.**
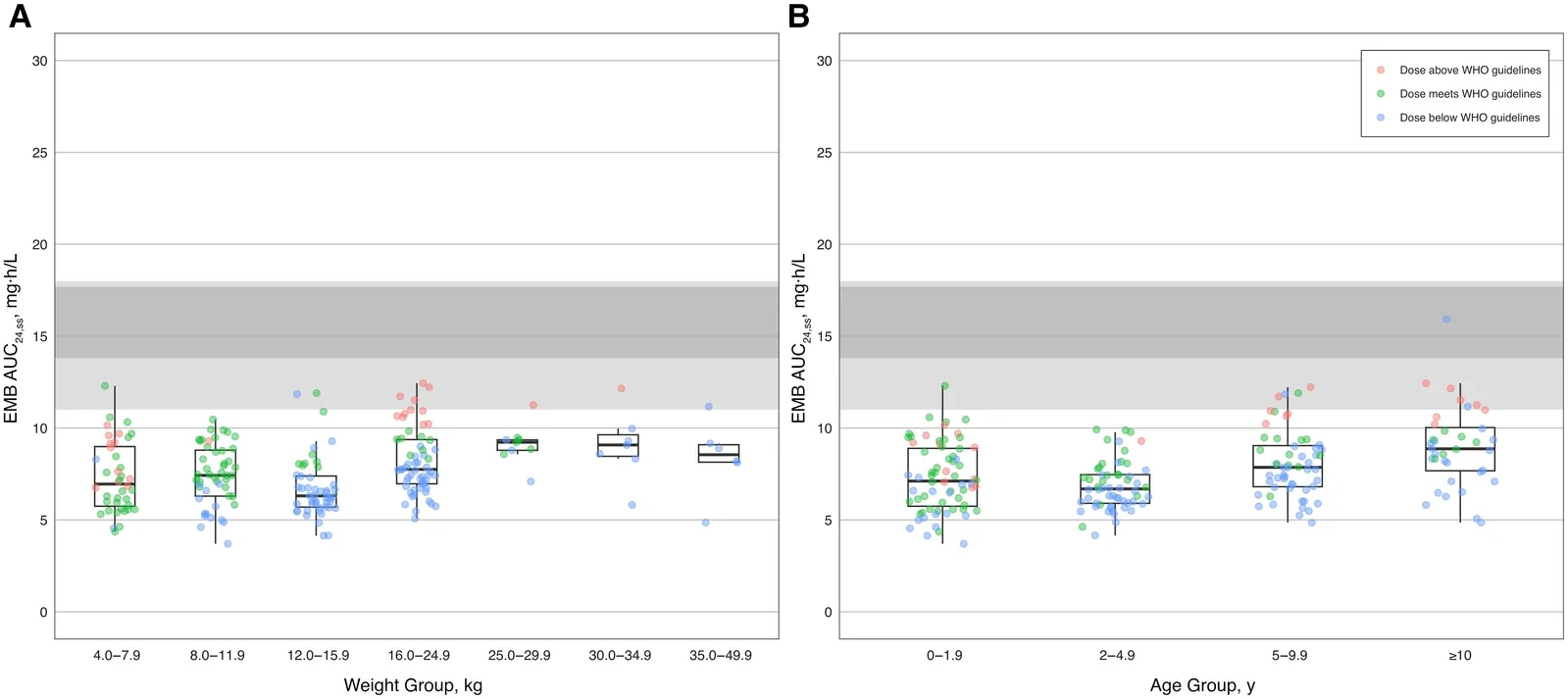
Observed ethambutol (EMB) 24-hour exposure (24-hour area under the curve at steady state [AUC_24,ss_]) for all pediatric participants across weight (*A*) and age (*B*) groups. Lighter and darker gray-shaded areas indicate the therapeutic window and the target exposure distribution, respectively. Abbreviation: WHO, World Health Organization.

The pcVPC plots on all 3 studies showed that most of the observed plasma concentrations of EMB were within the 95% prediction intervals, indicating little probability of model misspecification (Figure 1). Notably, the lower bound of the 95% confidence interval in the study by Bekker et al [13] was relatively wide, likely due to greater variability or a smaller sample size in that study. Furthermore, the median of the parameters estimated from bootstrapped datasets was comparable to the estimated value in the final model (Table 3). Collectively, the pcVPC plots and bootstrapping results supported model stability and good agreement between the model predictions and the observed data. With treatment according to the trial dosing regimen, 77.4% of the AUC_24,ss_ for participants was below the target exposure distribution, with even higher proportions observed in those weighing <12.0 kg (88.6%) or <5 years old (89.7%) (Figure 2).

### Optimal Dosing Simulations

Model-informed, optimized weight-band doses ranged from 100 to 550 mg, using either the 100- or 400-mg single-drug formulations or the 275-mg FDC of EMB, for participants weighing <45 kg (Table 4) were simulated. Compared to the current weight-band dosing, about 40.5% of the patients would have received a higher dose and 5% more patients may benefit from the optimized weight-band dosing (Supplementary Figure 9). Model-informed weight-band dosing resulted in a higher proportion of participants achieving concentrations within the target distribution of exposure, compared to the current weight-band dosing, across all weight bands and nutritional statuses (Supplementary Figures 9–12). Model-informed dosing recommendations were up to twice the current WHO-recommended dose for children weighing <25 kg, and as low as three-quarters the recommended dose for those >25 kg (Table 4).

**Table 4.** Currently Recommended and Optimized Pediatric Weight-Band Dosing for Ethambutol

Available Formulations	Weight Band, kg	Current Weight-Band WHO Dosing^a^		Harmonized Weight Band, kg	Model-Informed Optimized Weight-Band Dosing	
		Dose, mg	No. of Tablets		Dose, mg	No. of Tablets
Single-drug tablets (100 or 400 mg)	4 to <8	100	1 (100-mg)	3 to <6 (age <3 mo)	50	0.5 (100 mg)
	…	…	…	3 to <6 (age ≥3 mo)	100	1 (100 mg)
	8 to <12	200	2 (100 mg)	6 to <10 (age <6 mo)	150	1.5 (100 mg)
	…	…	…	6 to <10 (age ≥6 mo)	200	2 (100 mg)
	12 to <16	300	3 (100 mg)	10 to <15	300	3 (100 mg)
	16 to <25	400	1 (400 mg)	15 to <20	400	1 (400 mg)
	…	…	…	20 to <25	500	1 (100 mg) + 1 (400 mg)
FDC tablet (HRZE 75/150/400/275 mg) or single-drug tablet (100/400 mg)	25 to <30	550 FDC or 600	2 (FDC) or 1.5 (400 mg)	25 to <30	825	2 (FDC)
	30 to <35	825 FDC or 800	3 (FDC) or 2 (400 mg)	30 to <35	825	3 (FDC)
	35 to <40	1100 FDC or 1200	4 (FDC) or 3 (400 mg)	35 to <40	825	3 (FDC)
	40 to <45	1100 FDC or 1200	4 (FDC) or 3 (400 mg)	40 to <45	1100	3 (FDC)

Abbreviations: FDC, fixed-dose combination; HRZE, isoniazid, rifampicin, pyrazinamide, and ethambutol; WHO, World Health Organization.

^a^Based on WHO 2022 guidelines. Ethambutol (preferably in dispersible tablet form) should be added in the intensive phase for children with extensive disease, those living with human immunodeficiency virus (HIV), or those living in a setting where the prevalence of HIV or isoniazid resistance is high.

## DISCUSSION

**Figure 3.**
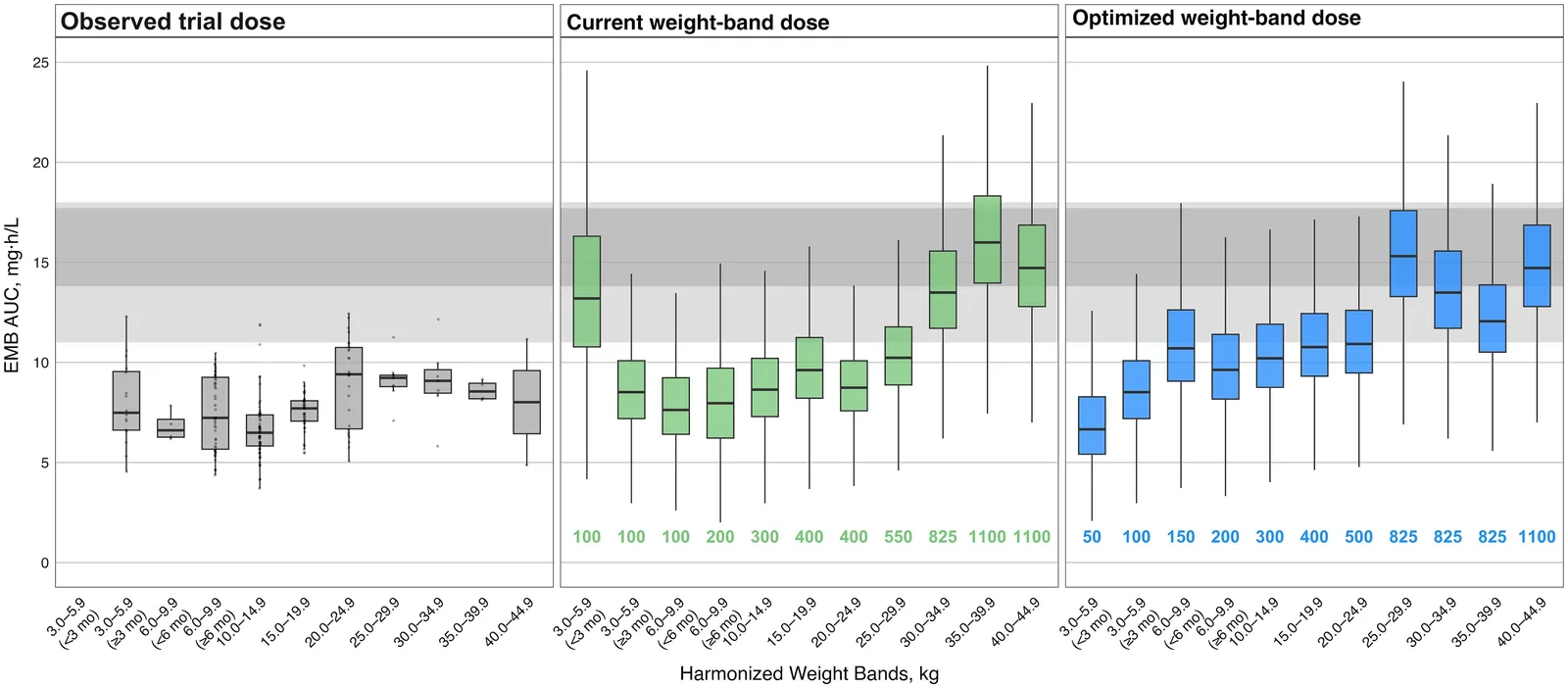
Observed 24-hour ethambutol (EMB) exposure (24-hour area under the curve at steady state [AUC_24,ss_], grey box plots) and simulated AUC_24,ss_ under the current World Health Organization (WHO)–recommended doses (green box plots) and model-informed optimized weight-band doses across harmonized weight bands (blue box plots). For the current weight-band box plots, the current WHO-recommended doses were used, but results are plotted according to harmonized weight bands. It was assumed that children weighing 3–4 kg received 100 mg under the current dosing regimen. Green and blue numbers indicate the minimum simulated dose for each harmonized weight band, and lighter and darker gray-shaded areas indicate the therapeutic window and the target exposure distribution, respectively.

In this study, we performed an IPDMA of the EMB PKs in children. EMB exposures in children receiving the current WHO-recommended doses were lower than the target exposure, particularly among those weighing <25 kg (Figure 3). Guided by the simulated exposures, we optimized the current weight-band doses by considering the availability of formulations, minimizing the number of tablets to facilitate administration, and addressing safety concerns. In addition, our population PK model incorporated age as a maturation function for CL to account for renal development in pediatric populations. This finding supports the conclusion that EMB CL in pediatric populations is largely influenced by age-dependent renal maturation. Given that EMB is primarily excreted renally, the maturation of renal function likely contributes to the observed increase in CL with age [28].

Our study showed that EMB exposure in children weighing <30 kg under the current WHO dosing strategy was substantially lower than the target exposure derived from adults. While EMB is used in combination with other first-line drugs for tuberculosis treatment, suboptimal EMB exposures may still be of concern, as they could contribute to reduced treatment efficacy or potentially increase the risk of drug-resistant tuberculosis or other serious adverse effects. Our results were consistent with those of other simulation studies, suggesting that the current regimen may result in underexposure in most pediatric participants [17–19, 25].

Although EMB is primarily eliminated unchanged via renal excretion and younger children may not yet have fully mature renal function, they can still exhibit lower EMB exposures when a weight-based dosing approach is applied, for 2 main reasons. First, the harmonized weight-band dosing proposes discrete dose increments rather than continuous milligram-per-kilogram dosing, which can lead to a lower milligram-per-kilogram dose at the upper end of a weight band. For example, patients weighing 3 to <6 kg (and <3 months of age) may receive 8.3–16.6 mg/kg, whereas those weighing 40 to <45 kg receive 24.4–27.5 mg/kg (Table 4). Second, age-dependent elimination may further reduce exposures in younger children due to faster drug CL and a relatively larger elimination organ–to–body size ratio [13, 15]. Together, dosing band effects and developmental physiology likely explains the lower EMB exposures observed in younger children under the current WHO harmonized weight-band dosing strategy.

Using model-informed dosing, the proportion of participants achieving target EMB exposure improved across all harmonized weight bands except for the weight bands of 3 to <6 kg (age <3 months) and 35 to <39.9 kg (Supplementary Figure 10). Compared with the current WHO dosing strategy, simulation results showed that the difference in the proportion of participants within the therapeutic windows was as high as 37.4% between current WHO dosing and model-informed weight-band dosing (Supplementary Figure 10). However, for children weighing <25 kg, the proposed dose is up to twice the current WHO recommendation, while for those >25 kg, it can be as low as three-quarters of the recommended dose (Table 4). Therefore, caution should be exercised when considering implementation of these revised doses. For example, optic neuropathy, the most well-recognized toxic effect of EMB, has been associated with higher exposures. Historically, the risk has often been described using dose-based thresholds, with increased incidence reported in adults receiving doses >25 mg/kg/day [29, 30]. However, available evidence suggests that toxicity may be more closely related to cumulative drug exposure, which is influenced by both the daily dose and the treatment duration [31].

Prior studies have shown that EMB-induced ocular toxicity increases in incidence with higher doses but can also occur at lower doses, indicating that no single dose threshold can be considered completely safe [31]. Although toxicity typically does not develop until after ≥1.5 months of treatment, participants may experience a sudden onset of decreased vision, which can occur despite careful ophthalmologic monitoring and is often permanent and irreversible [29, 32].

Our study also showed that both malnourished and well-nourished participants weighing <25 kg tended to show lower exposure to EMB when treated according to the current WHO dosing recommendation (Supplementary Figure 9). Notably, under the current WHO dosing approach, the median EMB exposure did not fall within the target exposure distribution across different and BMI-for-age *z* score groups (Supplementary Figure 11), suggesting that malnourished children may have received lower doses than appropriate. To address this issue, in addition to a model-informed weight-band dosing strategy, age-band dosing may serve as a potential alternative dosing, as it reduces reliance on assumptions regarding patients’ typical body weight (Supplementary Table 4) [3]. Our simulations demonstrated that participants with more severe malnutrition had a higher likelihood of achieving target exposure with age-band dosing (Supplementary Figure 11). This difference became less pronounced as the severity of malnutrition decreased. These findings indicate that age-band dosing may be a viable alternative in settings with a high prevalence of malnutrition among pediatric populations.

When administering treatment to younger adolescents weighing >25 kg, a single-drug formulation may be used, as is done in pediatric dosing, instead of FDC tablets (Table 4). However, using single-drug tablets may increase the risk of nonadherence, which may also lead to unfavorable outcomes. On the other hand, single-drug tablets allow for more flexible dose adjustments without the risk of overdosing or underdosing the other component drugs, such as isoniazid, rifampicin, and pyrazinamide (Table 4). Therefore, while FDC tablets remain preferable for ease of administration, single-drug tablets may be a reasonable alternative in specific cases where individualized dosing is necessary.

Although a revised harmonized weight-band EMB dosing table was proposed to optimize drug exposure, pill burden remains an important practical consideration, particularly in pediatric populations (Table 4). Under the current formulation, achieving the recommended dose often requires the administration of multiple tablets, which may negatively affect treatment adherence and acceptability. The development of new EMB formulations with age- or weight-appropriate strengths would substantially reduce pill burden, thereby simplifying dosing, improving adherence, and potentially enhancing treatment outcomes in children.

This study has 2 main limitations. First, the EMB IPDMA dataset lacked sufficient participants at the extremes of covariate distributions (eg, weight and age). Specifically, 14 participants (6.4%) were <6 months old and 10 (4.5%) weighed <5 kg, while 36 (16.4%) were >10 years old and 6 (2.7%) weighed >35 kg. Although this IPDMA dataset represents the largest pediatric dataset used to develop a population PK model for EMB, these subgroups remain underrepresented. Therefore, model-predicted exposures and dosing recommendations for these groups should be interpreted with caution. Second, patient-specific efficacy and safety outcome were not evaluated to directly support the model-based dosing recommendations. Future studies incorporating clinical end points are needed to validate and refine these strategies.

In conclusion, we believe that our IPDMA supports the revision of current pediatric EMB dosing guidelines for the treatment of tuberculosis, together with close monitoring for possible adverse effects. Our findings aim to ensure adequate drug exposure across all pediatric weight bands, while also accounting for toxicity and challenges related to formulation implementation and adherence.

## Supplementary Material

jiag194_Supplementary_Data

## Supplementary Data

Supplementary materials are available at *The Journal of Infectious Diseases* online (http://jid.oxfordjournals.org/). Supplementary materials consist of data provided by the author that are published to benefit the reader. The posted materials are not copyedited. The contents of all supplementary data are the sole responsibility of the authors. Questions or messages regarding errors should be addressed to the author.
