# Supplementary material for: Pharmacokinetics and Dose Optimization of Ethambutol in Children on First-Line Antituberculosis Therapy: An Individual Patient Data Meta-Analysis
Source: J Infect Dis. 2026 Mar 30;234(3):e613–22. doi: 10.1093/infdis/jiag194 (PMC13600441; doi:10.1093/infdis/jiag194)
Supplement: Supplementary file 1 [file jiag194_supplementary_data.docx]

Supplementary Materials:

**Pharmacokinetics and Dose Optimization of Ethambutol in Children on First-line Antituberculosis Therapy: An Individual Patient Data Meta-Analysis**

Tae Kyu Chung,^1,2^ Eunsol Yang,^1,2^ Maureen Shin,^1,2^ Belen P. Solans,^1,2^ Xujia Zhou,^1,2^ Sejung Hwang,^1,2^ Michael C. Yoon,^1,2^ Dvijen Purohit,^1,2^ Dongsheng Yang,^1,2^ Samer Mouksassi,^1,2^ Agathe Béranger,^1,2,3,4^ Adrie Bekker,^5^ Awewura Kwara,^6^ Navarat Panjasawatwong,^7^ Radojka M. Savic,^1,2*^

^1^ Department of Bioengineering and Therapeutic Sciences, Schools of Pharmacy and Medicine, University of California-San Francisco, San Francisco, California, USA

^2^ Center for Tuberculosis, University of California-San Francisco, San Francisco, California, USA

^3^ Université Paris Cité, INSERM, UMR1343, Pharmacologie et évaluation des thérapeutiques chez l'enfant et la femme enceinte, F-75006 Paris, France

^4^ Réanimation et Soins Intensifs Polyvalents Pédiatriques - SMUR pédiatrique, AP-HP.Centre, Hôpital Necker-Enfants malades, F-75015, Paris, France

^5^ Department of Paediatrics and Child Health, Stellenbosch University, Cape Town, South Africa

^6^ Division of Infectious Diseases and Global Medicine, University of Florida College of Medicine, Gainesville, Florida, USA

^7^ Department of Pharmaceutical Care, Faculty of Pharmacy, Payap University, Chiang Mai, Thailand

**Table of Contents**

[**Harmonized Weight Band**	1](#_Toc224734876)

[**Identification and Selection of Eligible Studies**	1](#_Toc224734877)

[Table S1. Study characteristics of pooled pediatric pharmacokinetic (PK) data considered for inclusion in individual participant data meta-analysis (IPDMA) for ethambutol.	2](#_Toc224734878)

[**Literature Review**	2](#_Toc224734879)

[Table S2. Previously developed population pharmacokinetic models of ethambutol in pediatric populations	1](#_Toc224734880)

[**Software**	2](#_Toc224734881)

[**Development of IPDMA Dataset and simulation dataset for Ethambutol**	2](#_Toc224734882)

[Figure S1. Distribution of patient characteristics in the IPDMA dataset	3](#_Toc224734883)

[Figure S2. Ethambutol pharmacokinetic profiles in the pediatric population included in the individual patient data meta-analysis dataset	3](#_Toc224734884)

[Figure S3. Observed vs expected weight-for-age in the ethambutol IPDMA dataset	4](#_Toc224734885)

[Figure S4. Distribution of malnutrition indicators of patients in the IPDMA dataset of ethambutol	5](#_Toc224734886)

[Figure S5. Weight versus age of patients included in the ethambutol simulation dataset. Red and blue dots represent individual patients from the Demographic and Health Surveys (DHS) Program and pediatric demographic data stored in-house from tuberculosis clinical trials	5](#_Toc224734887)

[**Target Range of Ethambutol**	6](#_Toc224734888)

[Table S3. Target exposures to ethambutol.	7](#_Toc224734889)

[**Base Model Development**	7](#_Toc224734890)

[**Covariate Model Development**	8](#_Toc224734891)

[**Final IPDMA Model Structure of Ethambutol**	8](#_Toc224734892)

[Figure S6. Final IPDMA model structure of ethambutol	9](#_Toc224734893)

[Figure S7. Estimated apparent clearance across (A) weight and (B) age ranges derived from the final model and previously developed population pharmacokinetic models of ethambutol	9](#_Toc224734894)

[Figure S8. General goodness-of-fit plots for the final population pharmacokinetic model of ethambutol	10](#_Toc224734895)

[**IPDMA Model Validation**	10](#_Toc224734896)

[**Ethambutol Exposure Outcomes**	11](#_Toc224734897)

[Figure S9. Simulated ethambutol exposure across harmonized weight band following current and optimized weight band dosing regimens stratified by nutritional status.	11](#_Toc224734898)

[**Proportion of target attainment following different dosing strategies**	13](#_Toc224734899)

[Table S4. Currently recommended weight band dosing and optimized pediatric age-band dosing for ethambutol	13](#_Toc224734900)

[Figure S10. Proportion of patients with ethambutol exposure within target exposure distribution, overdosed, and underdosed across harmonized weight bands.	14](#_Toc224734901)

[Figure S11. Simulated ethambutol exposure across body-mass-index (BMI)-for-age z score groups following current, optimized weight, and optimized age-band dosing regimens.	15](#_Toc224734902)

[Figure S12. Proportion of patients within the therapeutic window across (A) nutritional status and (B) severity of malnutrition, following the current, optimized weight-band, and optimized age-band dosing regimens.	16](#_Toc224734903)

[Control stream	17](#_Toc224734904)

[References	21](#_Toc224734905)

## **Harmonized Weight Band**

Harmonized weight bands across therapeutic areas have been proposed to improve the quality of care and simplify drug prescribing for children with comorbidities [1, 2]. We used our models to assess exposures to first-line tuberculosis drugs when applying these harmonized weight bands.

## **Identification and Selection of Eligible Studies**

We identified eligible studies that reported both clinical outcome and PK data for EMB in pediatric populations by searching the PubMed database, without restrictions on language or publication status, from January 2010 (when dosing recommendations were updated) to January 2022.

The search strategy used was: ("ethambutol"[All Fields] AND ("child*"[All Fields] OR "pediatric*"[All Fields] OR "paediatric*"[All Fields]) AND "pharmacokinetic*"[All Fields]).

Additional studies were also identified through direct collaborations with authors or manual searches of reference lists from included studies and review articles (Table S1). We screened abstracts and reviewed full articles to identify studies that enrolled at least 20 participants under 18 years old, had at least two PK samples per occasion, and involved EMB use for TB infection, TB disease, or preventive therapy. Studies were excluded if the treatment was unrelated to TB or lacked dosing information. Studies missing TB treatment outcome data were still included in the PK analysis.

All articles were screened and selected independently by two reviewers (A.B. and B.P.S.). Key studies were identified through consensus with a third reviewer (R.M.S.). Following the finalization of data use agreements, we contacted the corresponding authors of the eligible studies to transfer data. All included studies had obtained ethical approval from their local ethical review boards. The analysis followed the updated Preferred Reporting Items for a Systematic Review and Meta-analysis of Individual Participant Data guideline. The protocol of this study was registered in PROSPERO (CRD42021275526) [3]. A total of five studies were identified through the literature search, and three of these agreed to share individual patient data and were selected for inclusion in the final dataset (**Table S1)**.

### Table S1. Study characteristics of pooled pediatric pharmacokinetic (PK) data considered for inclusion in individual participant data meta-analysis (IPDMA) for ethambutol.

| **Study, year** | **Study country** | **Pediatric participants (n)** | **PK Observations (n)** |
| --- | --- | --- | --- |
| **Bekker et al., 2016^a^ [4]** | South Africa | 16 | Predose, H1, H2, H4, H6, and H8 post-dose |
| **Horita et al., 2018[5]** | Ghana | 113 | Week 4 or later:  Pre dose, H1, H2, H4, H8 |
| **Panjasawatwong et al., 2020[6]** | Vietnam | 100 | Day 1 and 14:  Two samples randomly drawn among H1, H2, H3, H4, H5, H6, H8, H12, H18, or H24  Day 30 and 90:  One sample randomly drawn among H3, H4, or H5 |
| Tikiso et al., 2022[7] | Malawi, South Africa, Zambia | 188 | Predose, H1, H2, H4, H6 and H10 post-dose |
| Hiruy et al., 2014[8] | South Africa | 13 | Predose, H0.42, H1.76, H3.37, H10.31, and H24 post-dose |
| **Bold** represents the studies that reached data sharing agreement and whose data that are used in this work.  ^a^Infant data from the DATiC trial. Bekker et al.’s study was also included in the Tikiso et al’s paper [7]. | | | |

## **Literature Review**

Population pharmacokinetic models of EMB were identified through a comprehensive literature search using the following medical subject headings (MeSH):

("Tuberculosis"[MeSH Terms] OR "tubercul*"[Title/Abstract] OR "TB"[Title/ Abstract] OR "TBC"[Title/Abstract]) AND ("Child"[MeSH Terms] OR "Infant"[MeSH Terms] OR "child, preschool"[MeSH Terms] OR "infant, newborn"[MeSH Terms] OR "child*"[Title/Abstract] OR "pediatr*"[Title/Abstract] OR "paediatr*"[Title/ Abstract] OR "infant*"[Title/Abstract] OR "baby"[Title/Abstract] OR "babies"[Title/ Abstract] OR "toddler*"[Title/Abstract] OR "kids"[Title/Abstract] OR "minors"[Title/Abstract] OR "newborn*"[Title/Abstract] OR "neonate*"[Title/ Abstract] OR "Adolescent"[MeSH Terms] OR "adolescen*"[Title/Abstract] OR "teen*"[Title/Abstract] OR "youth*"[Title/Abstract] OR "young"[Title/Abstract]) AND ("ethambutol"[MeSH Terms] OR "ethambutol"[All Fields]) AND "compartment"[All Fields]

Only models based on pediatric data collected since the publication of the first WHO childhood tuberculosis treatment guidelines (i.e., 2006 or later) were considered. As of April 2025, four population pharmacokinetic (PK) models of EMB in pediatric populations were identified. A detailed description of these previously developed population PK models is provided in **Table S2**, including information on study population, structural model, covariates included, and final parameter estimates.

### Table S2. Previously developed population pharmacokinetic models of ethambutol in pediatric populations

| **Descriptions** | **Studies included** |  |  |  |
| --- | --- | --- | --- | --- |
|  | **Hiruy et al. (2014)[8]** | **Horita et al. (2018)[5]** | **Panjasawatwong et al. (2020)[6]** | **Tikiso et al (2022)[7]** |
| Number of subjects | 13 | 48 | 100 | 188 |
| Number of concentrations | NR | 547 | 517 | 1012 |
| Structural model | 1 CMT | 2 CMT with zero order absorption, lag time, first order elimination | 2 CMT with 2-fixed transit absorption CMTs | 2 CMT with 4.82 transit absorption CMTs |
| Error model | NR | Proportional | Additive residual error (logarithmic scale) | Combination error |
| Covariates included | None | Body weight on CL, Q, Vc, & Vp | Body weight on CL, Q, Vc & Vp, maturation function on CL/F | Body weight on CL, Q, Vp, & Vc, maturation function on CL, lopinavir/ritonavir, break point for age, age on F, clinical studies on Ka |
| Software used | ADAPT5 | MonolixSuite2016R1 | NONMEM | NONMEM |
| **Primary PK parameter** | **Population estimate^*^ (%CV) [RSE]** | | | |
| T_lag_ (h) | - | 0.7 (17.5) [7.0] | - | - |
| T_k0_ (h) | - | 0.9 (66.5) [16.0] | - | - |
| F | 1, fixed | 1, fixed | 1, fixed (20.1) | 1, fixed (33.1, BOV) |
| CL/F (L/h) | 2.7 (NR) [NR] | 32.5 (48.3) [32.5] | 28.2 (0, fixed) [4.7] | 15.9 (11.6) [NR] |
| K_a_ (1/h) | 1.7 (NR) [NR] | - | - | 1.4 (63.7, BOV) [NR] |
| V_c_/F (L) | 135.0 (NR) [NR] | 112 (66.7) [112] | 98.6 (51.1) [11.0] | 44.3 [NR] |
| MTT (h) | - | - | 1.8 (18.4) [7.2] | 40.4 (48.5, BOV) [NR] |
| MAT50 (mo) | - | - | 4.0 (0 fixed) [36.0] | - |
| PMAGE50 (mo) | - | - | - | 10.8 [NR] |
| Hill | - | - | 1, fixed | 3.3 [NR] |
| Q/F (L/h) | - | 15.4 (27.9) [10.0] | 16.9 (12.2) [NR] | 11.5 [NR] |
| V_p_/F (L) | - | 97.8 (31.8) [8.0] | 153.0 (96.3) [16.0] | 86.2 [NR] |
| BW on CL/F |  | 0.4, fixed [24.0] | 0.75, fixed | 0.75, fixed |
| BW on V_c_/F |  | 0.2 (66.7) [7.0] | 1, fixed | 1, fixed |
| BW on Q/F |  | 97.8 (31.8) [10.0] | 0.75, fixed | 0.75, fixed |
| BW on V_p_/F |  | 0.9, fixed [60.0] | 1, fixed | 1, fixed |
| Change in oral bioavailability  post-ARM | - | - | - | - |
| Num of ATC | - | - | - | 4.8 (0.0) [NR] |
| Breakpoint for age effect on F | - | - | - | 3.2 [NR] |
| Change in speed of absorption in DNDi and SHINE (%) | - | - | - | -23.6 [NR] |
| Change in F when on LPV/r (%) | - | - | - | -32.0 [NR] |
| Age on F | - | - | - | 0.1 [NR] |
| Scaling of BOV in F | - | - | - | 1.4 [NR] |
| Additive error | - | - | 0.2 (log transformed) (4.5) [NR] | 16.8, fixed (-) [NR] |
| Proportional error | - | 0.3 [6.0] | - | 17.7 (-) [NR] |
| ATC, absorption transit compartment; BW, body weight; % CV, coefficient of variation expressed in %; CL/F, apparent clearance; CMT, compartment; F, bioavailability; Hill, Hill coefficient for maturation clearance; IIV, interindividual variability; LPV/r, lopinavir/ritonavir; MAT50, postmenstrual age at which clearance is 50% of the mature clearance; MTT, mean transit absorption time; NR, not reported; T_lag_, lag time; Vc/F, apparent volume of distribution in central compartment; PMAGE50, postmenstrual age at which 50% of the maturation is complete; V_p_/F, apparent volume of distribution in peripheral compartment; Q/F, intercompartmental clearance | | | | |

## **Software**

NONMEM (version 7.5; Icon Development Solution, Gaithersburg, MD) was used for population nonlinear mixed effects PK modeling, with the first order conditional estimation method with interaction (FOCE-I) employed for parameter estimation. R v.4.4.1 (R Foundation, Vienna, Austria) was used for graphical presentations and exposure calculations. Xpose (version 4.7.3) [9] and the PsN toolkit (version 5.3.0) [10] were used for generating diagnostic plots including pcVPC and bootstrapping.

## **Development of IPDMA Dataset and simulation dataset for Ethambutol**

Continuous covariates included age, body weight, height, body mass index (BMI), and anthropometric z-scores—specifically, weight-for-age (WAZ), height-for-age (HAZ), weight-for-height (WHZ), and BMI z-scores (BAZ), based on WHO reference standards [11]. Categorical covariates included sex, race, HIV status, nutritional status (malnourished vs. well-nourished; Development of IPDMA Dataset for Ethambutol section & Figure S4 [12]), and administration route (oral vs. nasogastric tube).

The dataset used for EMB model development had a median patient age of 4.0 years, height of 48.0 cm, and weight of 12.5 kg (Figure S1). The exposure to EMB appeared to differ across weight groups (Figure S2).

Figure S1. Distribution of patient characteristics in the IPDMA dataset

Figure S2. Ethambutol pharmacokinetic profiles in the pediatric population included in the individual patient data meta-analysis dataset. Colored circles represent all observed ethambutol concentrations, including both Day 1 and steady-state data, plotted together. The black solid line represents the median observed ethambutol concentrations for the entire population, while the three colored dashed lines represent the median concentrations for each weight group.

The population PK dataset for EMB included underweight patients (Figure S3), ensuring the model captured variability associated with nutritional status in pediatric tuberculosis populations. WAZ (for children up to 5 years old) and BAZ scores (for children up to 10 years old) were selected to assess the effect of the maturation function on population PK model parameters, as WAZ is an indicator of malnutrition in children up to 5 years old, and less than 2.5% of BAZ data were missing for patients aged 10 years and under (Figure S4). As a result, malnourished participants were defined as patients with WAZ below -2.0 for age under 5 years old, BAZ below -2 for age between 5 and 10 years old, and BMI below 18.5 for age above 10 years old [12].

### Figure S3. Observed vs expected weight-for-age in the ethambutol IPDMA dataset

Figure S4. Distribution of malnutrition indicators of patients in the IPDMA dataset of ethambutol

Figure S5. Weight versus age of patients included in the ethambutol simulation dataset. Red and blue dots represent individual patients from the Demographic and Health Surveys (DHS) Program and pediatric demographic data stored in-house from tuberculosis clinical trials [13], respectively. The DHS dataset included patients up to 5 years of age. Blue lines with shaded areas indicate the median weight-for-age z-scores and their 95% confidence intervals. Brown and dark blue lines represent the Locally estimated scatterplot smoothing for each data source.

## **Target Range of Ethambutol**

The exposure target for dose optimization was defined as the therapeutic window and target distribution established in adults from the TBTC Study 31/A5349 (S31), the largest and most diverse phase III clinical study to date for tuberculosis medications, including a population from 13 countries and a sample size of 819 individuals [14].

The therapeutic window was defined as the range of EMB exposure associated with at least 95% cure and less than 20% incidence of any grade 3-5 adverse events in S31 (Table S3, Alkhafaji et al., manuscript in preparation). The target distribution was defined as the 25th–75th percentile exposure range for ethambutol observed in Study 31 (S31)[15]. Proportion of target attainment was calculated based on the target exposure distribution (Table S3).

### Table S3. Target exposures to ethambutol.

| **Drug** | **Exposure target to ethambutol (AUC_ss, target_)** |
| --- | --- |
| Therapeutic window ($mg\cdot h/L)$ | 11.0 – 18.0 |
| Target exposure distribution ($mg\cdot h/L)$[15] | 13.8–17.7 |

AUC_ss, target_ = target area under the concentration time curve at steady state over 24 hours

## **Base Model Development**

One- and two-compartment models were evaluated with first-order absorption and both linear and non-linear elimination. Inter-individual variability (IIV), inter-occasion variability (IOV), and inter-study variability (ISV) for the PK parameters were estimated using an exponential error model (Equation 1):

$P_{i}=P_{p}\cdot\exp\left( {\eta_{P}}_{i} \right)\cdot exp({\eta_{P}}_{ij})\cdot exp({\eta_{P}}_{k})$	Equation 1

In this equation, $P_{i}$ is the PK parameter for i^th^ individual, $P_{p}$ is the typical population value of PK parameter, ${\eta_{P}}_{i}$ represents the IIV for the PK parameter in the *i*^th^ individual, ${\eta_{P}}_{ij}$ represents the IOV for the PK parameter in the *i*^th^ individual at the *j*^th^ occasion, and ${\eta_{P}}_{k}$ represents the ISV for the PK parameter in *k* studies. The estimated IIV, IOV, and ISV were assumed to follow a log-normal distribution with a mean of 0 and a variance of $\omega^{2}$. Additive, proportional and combined proportional-additive error models were tested. To account for the delay in absorption phase, models incorporating either an absorption lag-time (ALAG1) or different numbers of fixed transit compartments were evaluated.

Throughout the model development process, model selection was guided by a decrease in the objective function value (OFV), reduction in the inter-individual variability (IIV) of the PK parameters, visual assessments of diagnostic plots, and the physiological and clinical plausibility of parameter estimates.

## **Covariate Model Development**

The effects of continuous covariates on PK parameters were evaluated using Equation 2:

$P_{i,cov}=P_{p}\cdot\left[ \frac{X_{i}}{median \left[ X \right]} \right]^{\theta_{cov}}$	Equation 2

In this equation, $P_{i,cov}$ represents the PK parameter for the *i*^th^ individual after incorporating the covariate effect, and $\theta_{cov}$ is the estimated covariate effect on the typical population value ($P_{p}$).

Covariates were added one at a time to the base population PK model and retained if the OFV decreased by greater than 3.84 ($p<0.05 for df=1$). The population PK model was then refined by sequentially removing covariates and IIV terms on PK parameters, one at a time. Covariates and IIV terms were reintroduced into the model if the OFV increased by greater than 6.64 ($p<0.01 for df=1$).

## **Final IPDMA Model Structure of Ethambutol**

The final model structure of EMB consisted of a two-compartment model with one transit compartment for the absorption (Figure S6). Compared to the one-compartment model without a transit compartment, the two compartment model reduced the objective function value (OFV) by 46.0. In addition, adding one fixed transit compartment further reduced the OFV by 34.8. This model adequately captured the absorption, distribution, and elimination characteristics observed in the dataset and provided a robust framework for simulating drug exposures across diverse pediatric subpopulations. The proposed model aligned well with previously published EMB models (Table S2). When the clearance (CL) equations were plotted, the CL estimates from our model were comparable to those reported by Panjasawatwong et al (Table S2 & Figure S7). In addition, our CL estimates lay in the middle of the range reported by other authors (Figure S7).

### Figure S6. Final IPDMA model structure of ethambutol

CL, clearance; Ktr, rate constant that governs the movement of the drug from the absorption site through the transit compartment and to the central compartment; Q, intercompartment clearance of unbound drug; Vc, volume of distribution in the central compartment; Vp, volume of distribution in the peripheral compartment.

### Figure S7. Estimated apparent clearance across (A) weight and (B) age ranges derived from the final model and previously developed population pharmacokinetic models of ethambutol

Figure S8. General goodness-of-fit plots for the final population pharmacokinetic model of ethambutol: observed vs. individual predicted plasma ethambutol concentrations (upper left); observed vs. population predicted plasma ethambutol concentrations (upper right); conditional weighted residuals (CWRES) vs. population predicted plasma ethambutol concentrations (lower left); CWRES vs. time since last dose (lower right)

## **IPDMA Model Validation**

No discernible bias was observed in any of the GOF plots (Figure S8). The model predictions aligned well with the observed data across the range of concentrations and time points, supporting the adequacy of the structural model and its parameter estimates. This suggests that the model provides a reliable description of EMB pharmacokinetics in the pediatric population.

## **Ethambutol Exposure Outcomes**

Compared to the current weight-band dosing, about 40.5% of the patients would have received a higher dose and 5% points more patients may benefit from the optimized weight-band dosing (Figure S9). Yet, only 10% of malnourished patients may attain target exposure even with the optimized weight band dosing. This stresses out that alternative dosing method that is age band dosing can further benefit these patients.

### Figure S9. Simulated ethambutol exposure across harmonized weight band following current and optimized weight band dosing regimens stratified by nutritional status.

Malnourished patients were defined as participants who has WAZ < -2 for children under 5 years of age and BAZ < -2 for those aged 5 years or older. Well-nourished patients were defined as participants who has WAZ ≥ -2 for children under 5 years of age and BAZ ≥ -2 for those aged 5 years or older. The lighter and darker grey shaded areas indicate the therapeutic window and the target exposure distribution, respectively.

When following the suggested age-band dosing method (Table S4), simulation experiments indicated improved EMB target exposure attainment in all pediatric populations as well as those with poor nutritional status (Figures S10-S12). Particularly, low weight children (BAZ < 0) consistently exhibited low AUC_ss_ with the current dosing method (Figure S10). In contrast, the proposed age-band dosing achieved more targeted EMB exposure across both age and nutritional status (Figures S10). Even when stratified by nutritional status, and severity of malnutrition, the proposed dosing method remained adequate across all categories (Figures S11). In conclusion, age-band dosing method led to a higher percentage of the pediatric population achieving therapeutic drug exposure, thereby potentially enhancing treatment efficacy and reducing the risk of subtherapeutic exposure, particularly in the most vulnerable age group. For convenience, the age-band dosing method might be a suitable alternative for treating patients with drug-sensitive tuberculosis.

## **Proportion of target attainment following different dosing strategies**

### Table S4. Currently recommended weight band dosing and optimized pediatric age-band dosing for ethambutol

| **Available formulations** | **Weight-band (kg)** | **Current weight-band World Health Organization dosing^a^** | | **Optimized age-band (yr)** | **Model-informed optimized age-band dosing** | |
| --- | --- | --- | --- | --- | --- | --- |
|  |  | HIV (+) | No. of tab. |  | HIV (+) | No. of tab. |
| Single tab. (100/400 mg) | 4 – < 8 | 100 mg | 1 x 100mg | 0.25 – < 1 | 100 mg | 1 x 100 mg |
|  | 8 – < 12 | 200 mg | 0.5 x 400 mg | 1 – < 2 | 200 mg | 2 x 100 mg |
|  | 12 – < 16 | 300 mg | [1 x 100 mg +  0.5 x 400 mg] | 2– < 5 | 300 mg | 3 x 100 mg |
|  | 16 – < 25 | 400 mg | 1 x 400mg | 5 – < 8 | 400 mg | 1 x 400 mg |
|  | - | - | - | - | - | - |
| HRZE tab. (75/150/400/275 mg)  SIngle tab. (400 mg) | 25 – < 30 | 550 (HRZE) or  600 mg | 2 HRZE or  1.5 x 400 mg | 8 – < 15 | 550 mg | 2HRZE |
|  | 30 – < 35 | 825 (HRZE) or  800 mg | 3 HRZE or  2 x 400 mg | - | - | - |
|  | 35 – < 40 | 1100 (HRZE) or  1200 mg | 4 HRZE or  3 x 400 mg | - | - | - |
|  | 40 – < 45 | 1100 (HRZE) or  1200 mg | 4 HRZE or  3 x 400 mg | - | - | - |
| ^a^Based on World Health Organization 2022 guidelines  Ethambutol (preferably using a dispersible tablet) should be added in the intensive phase for children with extensive disease, or if living with HIV, or if living in a setting where the prevalence of HIV or of isoniazid resistance is high. Our simulated dataset included few patients under 0.25 years of age; therefore, this age group was excluded from the analysis. | | | | | | |

### Figure S10. Proportion of patients with ethambutol exposure within target exposure distribution, overdosed, and underdosed across harmonized weight bands.

### Figure S11. Simulated ethambutol exposure across body-mass-index (BMI)-for-age z score groups following current, optimized weight, and optimized age-band dosing regimens.

Pharmacokinetic profiles were summarized as area under the concentration time curve at steady state (AUC_ss_) for ethambutol. The lighter and darker grey shaded areas indicate the therapeutic window and the target exposure distribution, respectively.

### Figure S12. Proportion of patients within the therapeutic window across (A) nutritional status and (B) severity of malnutrition, following the current, optimized weight-band, and optimized age-band dosing regimens.

Well-nourished children were defined as those with weight-for-age, weight-for-height, height-for-age, and body mass index-for-age z-scores (WAZ, WHZ, HAZ, and BAZ) ≥ 0. Children were considered underweight, stunted, and wasted if WAZ < -2, HAZ < -2, and WHZ < -2, respectively. The severity of malnutrition was defined by the WAZ: Not malnourished (WAZ ≥ 0), mildly malnourished (-2 ≤ WAZ < 0), moderately malnourished (-3 ≤ WAZ < -2), and severely malnourished (WAZ < -3).

# Control stream

;; 1. Based on: run650

;; 2. Description: 2CMT_prop 1MTT_ allometic_CL,Q,V,VP, MF_Fixed_reestimated_final

;; x1. Author: user

;; 3. Label: AMT: mg, TIME: hr; DV:

;; 1. IPDMA EMB model

;; Author: Tae Chung

$PROBLEM	EMB_ipdma_peds model

$INPUT	C STUDY ID RID USUBJIDN=DROP FIRSTDDATE=DROP ODATE=DROP NEWTIME=DROP DAY=DROP

DV DV2 LNDV2 MDV EVID AMT AMT2 ADDL II CMT LLOQ BLOQ ACTTIME=DROP TAF=DROP

SITE AFRICAN ARM AGE SEX RACE HIV CD4 VIRALLOAD HT WT

BMI CAVITY CXRCLASS=DROP CXREXTNT=DROP SMEAR=DROP KARNOFSKY=DROP AE_SAE DIABETES

ALCOHOL DOSEESTX DTC NDOT VOMIT SEVMAL DRUG TIME TAD NO_PK=DROP DOSEE

TRAIN=DROP PEDIATRIC DIABETESNEW=DROP HAZ WAZ WHZ BAZ MALN OCC EXWT

$DATA	NMdata_EMB_adults_peds_combined_final11.csv IGNORE=@ IGNORE=(STUDY.LE.9) IGNORE=(STUDY.EQ.31) IGNORE=(BLOQ.EQ.1)

$SUBROUTINE ADVAN13 TOL=3

$MODEL	NCOMP=5

COMP=(ABS DEFDOSE)

COMP=(CENT DEFOBS)

COMP=(PERI)

COMP=(TRAN)

COMP=(AUC) ; AUC compartment

$PK

;Interoccasional variability

;IF (OCC.EQ.1)	IOV_CL = ETA(7)

;IF (OCC.EQ.2)	IOV_CL = ETA(8)

;IF (OCC.EQ.1)	IOV_MTT= ETA(9)

;IF (OCC.EQ.2)	IOV_MTT= ETA(10)

IF (OCC.EQ.1)	IOV_F = ETA(7)

IF (OCC.EQ.2)	IOV_F = ETA(8)

MF_CL = (PMA**HILL)/(TM50**HILL + PMA**HILL)

;Equation required for covariate effects

STWT	= (WT/12.5) ; Median WT: 12.5 kg

PMA = ((AGE+0.001)*52) + 40

HILL = THETA(11)

TM50 = THETA(12)

;; Malnourished_y/n

; (THETA)**MALN

;; Severity of malnourishment

;IF (SEVMAL.EQ.0) MAL = 1

;IF (SEVMAL.EQ.1) MAL = (1 - THETA(15))

;IF (SEVMAL.EQ.2) MAL = (1 - THETA(16))

;IF (SEVMAL.EQ.5) MAL = (1 - THETA(16))

;IF (SEVMAL.EQ.3) MAL = (1 - THETA(17))

;; Sex effect

;IF (SEX.EQ.1) SEXCL = 0

;IF (SEX.EQ.0) SEXCL = 1

;; Race effect

;IF (RACE.EQ.2)	RACEEFF = 1

;IF (RACE.EQ.1)	RACEEFF = (1 + THETA(15))

;IF (RACE.EQ.5)	RACEEFF = (1 + THETA(16))

TVCL	= THETA(1)

CL	= TVCL	*EXP(ETA(1))	* (STWT **THETA(9)) * MF_CL

TVVC	= THETA(2)

VC	= TVVC	*EXP(ETA(2))	* (STWT **THETA(10))

;TVKA	= THETA(3)

;KA	= TVKA	*EXP(ETA(3))

TVMTT	= THETA(3)

MTT	= TVMTT	*EXP(ETA(3))

KTR	= 2/MTT

TVQ	= THETA(4)

Q	= TVQ	*EXP(ETA(4))	* (STWT **THETA(9))

TVVP	= THETA(5)

VP	= TVVP	*EXP(ETA(5))	* (STWT **THETA(10))

TVF1	= THETA(6)

F1	= TVF1	*EXP(ETA(6) + IOV_F)

;TVALAG = THETA(6)

;ALAG1	= TVALAG1	*EXP(ETA(6))

K14 = KTR

K42 = KTR

K20 = CL/VC

K23 = Q/VC

K32 = Q/VP

S2 = VC ;cmp 2 = central compartment

$DES

DADT(1) = -K14*A(1)

DADT(2) = K42*A(4) - K23*A(2) + K32*A(3) - K20*A(2)

DADT(3) = K23*A(2) - K32*A(3)

DADT(4) = K14*A(1) - K42*A(4)

DADT(5) = A(2)/VC

$THETA

(0, 30.3) ; 1 CL L/hr

(0, 61.3) ; 2 VC L

(0, 1.98) ; 3 MTT

(0, 23.2) ; 4 Q L/hr

(0, 148) ; 5 VP L

(1) FIX ; 6 F1

(0, 0.359) ; 7 prop error

(0) FIX ; 8 additive error

(0.75) FIX ; 9 wt on cl

(1) FIX ; 10 wt on v

(3.25) FIX ; 11 Hill

(0, 42.1) ; 12 PMA50

$OMEGA

0.0372 ; 1 CL IIV

0.51 ; 2 V IIV

0.0488 ; 3 MTT

0 FIX ; 4 Q

0 FIX ; 5 VP

0 FIX ; 6 F1

$OMEGA BLOCK(1)

0.159 ; IOV_F1

$OMEGA BLOCK(1) SAME

$SIGMA 1 FIX

$ERROR

AUC	= A(5)

IPRED	= A(2)/VC

PROP	= THETA(7)

ADD	= THETA(8)

W	= SQRT(PROP**2*IPRED**2 + ADD**2)

IRES	= DV-IPRED

IWRES	= IRES/W

Y = IPRED + W*EPS(1)

$ESTIMATION NOABORT PRINT=5 MAXEVAL=9999 METHOD=1 INTERACTION POSTHOC

SIGL=3 NSIG=1

$COVARIANCE PRINT=E UNCONDITIONAL

; Xpose

$TABLE NOPRINT ONEHEADER ID TIME OCC TAD AMT CMT MDV IPRED IWRES CWRES FILE=sdtab651

$TABLE NOPRINT ONEHEADER ID TIME OCC TAD CL VC KTR ETA1 ETA2 ETA3 FILE=patab651

$TABLE NOPRINT ONEHEADER ID CL WT HT BMI AGE HAZ WAZ WHZ BAZ FILE=cotab651

$TABLE NOPRINT ONEHEADER ID OCC STUDY DOSEE SEX RACE HIV SEVMAL MALN PEDIATRIC FILE=catab651

# References

1.	Galileya LT, Wasmann RE, Chabala C, et al. Evaluating pediatric tuberculosis dosing guidelines: A model-based individual data pooled analysis. PLoS Med **2023**; 20(11): e1004303.

2.	Lin YJ, van der Laan LE, Karlsson MO, Garcia-Prats AJ, Hesseling AC, Svensson EM. Model-Informed Once-Daily Dosing Strategy for Bedaquiline and Delamanid in Children, Adolescents and Adults with Tuberculosis. Clin Pharmacol Ther **2025**; 117(5): 1292-302.

3.	Agathe Beranger RS, Ryo Miyakawa, Belén Pérez Solans. Population pharmacokinetics of first-line antituberculosis drugs (rifampicin, isoniazid, pyrazinamide and ethambutol) in children treated for drug-susceptible tuberculosis: Individual patient data meta-analysis. Available at: <https://www.crd.york.ac.uk/PROSPERO/view/CRD42021275526>.

4.	Bekker A, Schaaf HS, Draper HR, et al. Pharmacokinetics of Rifampin, Isoniazid, Pyrazinamide, and Ethambutol in Infants Dosed According to Revised WHO-Recommended Treatment Guidelines. Antimicrob Agents Chemother **2016**; 60(4): 2171-9.

5.	Horita Y, Alsultan A, Kwara A, et al. Evaluation of the Adequacy of WHO Revised Dosages of the First-Line Antituberculosis Drugs in Children with Tuberculosis Using Population Pharmacokinetic Modeling and Simulations. Antimicrob Agents Chemother **2018**; 62(9).

6.	Panjasawatwong N, Wattanakul T, Hoglund RM, et al. Population Pharmacokinetic Properties of Antituberculosis Drugs in Vietnamese Children with Tuberculous Meningitis. Antimicrob Agents Chemother **2020**; 65(1).

7.	Tikiso T, McIlleron H, Abdelwahab MT, et al. Population pharmacokinetics of ethambutol in African children: a pooled analysis. J Antimicrob Chemother **2022**; 77(7): 1949-59.

8.	Hiruy H, Rogers Z, Mbowane C, et al. Subtherapeutic concentrations of first-line anti-TB drugs in South African children treated according to current guidelines: the PHATISA study. J Antimicrob Chemother **2015**; 70(4): 1115-23.

9.	Keizer RJ, Karlsson MO, Hooker A. Modeling and Simulation Workbench for NONMEM: Tutorial on Pirana, PsN, and Xpose. CPT Pharmacometrics Syst Pharmacol **2013**; 2(6): e50.

10.	Lindbom L, Pihlgren P, Jonsson EN. PsN-Toolkit--a collection of computer intensive statistical methods for non-linear mixed effect modeling using NONMEM. Comput Methods Programs Biomed **2005**; 79(3): 241-57.

11.	Organization WH. WHO child growth standards: length/height-for-age, weight-for-age, weight-for-length, weight-for-height and body mass index-for-age: methods and development. WHO child growth standards: length/height-for-age, weight-for-age, weight-for-length, weight-for-height and body mass index-for-age: methods and development, **2006**.

12.	Radtke KK, Dooley KE, Dodd PJ, et al. Alternative dosing guidelines to improve outcomes in childhood tuberculosis: a mathematical modelling study. Lancet Child Adolesc Health **2019**; 3(9): 636-45.

13.	ICF. The DHS Program. Available at: <https://dhsprogram.com/Data/> (accessed December 20 2018).

14.	Dorman SE, Nahid P, Kurbatova EV, et al. High-dose rifapentine with or without moxifloxacin for shortening treatment of pulmonary tuberculosis: Study protocol for TBTC study 31/ACTG A5349 phase 3 clinical trial. Contemp Clin Trials **2020**; 90: 105938.

15.	Chang VK, Imperial MZ, Phillips PPJ, et al. Risk-stratified treatment for drug-susceptible pulmonary tuberculosis. Nat Commun **2024**; 15(1): 9400.
